# Risks and benefits of percutaneous coronary intervention in spontaneous coronary artery dissection

**DOI:** 10.1136/heartjnl-2020-318914

**Published:** 2021-05-18

**Authors:** Deevia Kotecha, Marcos Garcia-Guimaraes, Diluka Premawardhana, Dario Pellegrini, Clare Oliver-Williams, Vasiliki Bountziouka, Alice Wood, Nalin Natarajan, Robert Jackson, Nathan Chan, Jan Ziaullah, Roby D Rakhit, Stephen P Hoole, Tom W Johnson, Jacek Kadziela, Peter Ludman, Nilesh J Samani, Angela H E M Maas, Robert-Jan van Geuns, Fernando Alfonso, David Adlam

**Affiliations:** 1 Department of Cardiovascular Sciences and NIHR Biomedical Research Centre, University of Leicester, Leicester, Leicestershire, UK; 2 Department of Cardiology, Hospital Universitario de La Princesa, Instituto de Investigación Sanitaria Princesa (IIS-IP), Universidad Autónoma de Madrid, CIBER-CV, Madrid, Spain; 3 Department of Cardiology, Hospital del Mar, Barcelona, Spain; 4 Department of Cardiology, Radboud University Medical Center, Nijmegen, The Netherlands; 5 Department of Cardiology, ASST Papa Giovanni XXIII, Bergamo, Italy; 6 Department of Biostatistics, University of Leicester, Leicester, Leicestershire, UK; 7 Department of Cardiology, Royal Free Hampstead, London, UK; 8 Royal Papworth Hospital and NIHR Cambridge Biomedical Research Centre, Cambridge, UK; 9 University Hospitals Bristol and Weston NHS Foundation Trust, Bristol, UK; 10 Department of Interventional Cardiology and Angiology, National Institute of Cardiology, Warsaw, Poland; 11 Institute of Cardiovascular Sciences, University of Birmingham, Birmingham, UK

**Keywords:** percutaneous coronary intervention, acute coronary syndrome

## Abstract

**Objective:**

To investigate percutaneous coronary intervention (PCI) practice in an international cohort of patients with spontaneous coronary artery dissection (SCAD). To explore factors associated with complications and study angiographic and longer term outcomes.

**Methods:**

SCAD patients (n=215, 94% female) who underwent PCI from three national cohort studies were investigated and compared with a matched cohort of conservatively managed SCAD patients (n=221).

**Results:**

SCAD-PCI patients were high risk at presentation with only 8.8% undergoing PCI outside the context of ST-elevation myocardial infarction/cardiac arrest, thrombolysis in myocardial infarction (TIMI) 0/1 flow or proximal dissections. PCI complications occurred in 38.6% (83/215), with 13.0% (28/215) serious complications. PCI-related complications were associated with more extensive dissections (multiple vs single American Heart Association coronary segments, OR 1.9 (95% CI: 1.06–3.39), p=0.030), more proximal dissections (proximal diameter per mm, OR 2.25 (1.38–3.67), p=0.001) and dissections with no contrast penetration of the false lumen (Yip-Saw 2 versus 1, OR 2.89 (1.12–7.43), p=0.028). SCAD-PCI involved long lengths of stent (median 46mm, IQR: 29–61mm). Despite these risks, SCAD-PCI led to angiographic improvements in those with reduced TIMI flow in 84.3% (118/140). Worsening TIMI flow was only seen in 7.0% (15/215) of SCAD-PCI patients. Post-PCI major adverse cardiovascular and cerebrovascular events (MACCE) and left ventricular function outcomes were favourable.

**Conclusion:**

While a conservative approach to revascularisation is favoured, SCAD cases with higher risk presentations may require PCI. SCAD-PCI is associated with longer stent lengths and a higher risk of complications but leads to overall improvements in coronary flow and good medium-term outcomes in patients.

## Introduction

Spontaneous coronary artery dissection (SCAD) is an important cause of acute coronary syndromes (ACS), predominantly affecting young to middle-aged women.^1–3^ Percutaneous coronary intervention (PCI) in acute SCAD has been associated with an increased risk of procedural complications compared with atherosclerotic ACS, with ‘PCI technical failure’ reported in 36%–53% of cases.^4 5^ Complications include iatrogenic dissection, haematoma extension and emergency coronary artery bypass grafting (CABG).^4–6^ When managed conservatively, most SCAD heals completely with restoration of apparently normal coronary architecture at follow-up.^7 8^ For this reason, the American and European Consensus Statements on SCAD both advocate conservative management over PCI, where this is possible.^1 2^

Accepting that conservative management is favoured ‘where possible’, there remains a significant population of SCAD patients where conservative management risks extensive infarction.^9 10^ For these higher risk cases, it remains unclear to what extent complications are a necessary cost of improving coronary perfusion and if a more proactive approach to revascularisation may be important for selected patient groups.

In this study, our objective was to investigate outcomes in a large observational SCAD-PCI cohort derived from three national registries. In particular, to investigate the balance of risks and benefits of PCI in a SCAD population.

## Methods

### Study population

The study was conducted in accordance with the Declaration of Helsinki. Patients and the public (http://beatscad.org.uk/) were involved in the funding, concept and dissemination of the study findings. Patients were recruited from the UK, Dutch and Spanish SCAD registries with SCAD events from 2003 to 2019. More details of these registries are provided in the online supplemental data. Patients in all studies gave written informed consent to data collection and contact for assessment of outcomes.

10.1136/heartjnl-2020-318914.supp1 Supplementary data

### Definition of PCI

PCI was defined by an apparent angiographic intent to intervene through, at minimum, the passage of a coronary guidewire except where guidewire passage was solely for the purpose of diagnostic intracoronary imaging.

All SCAD patients with PCI were included. To fully characterise SCAD in the PCI cohort, each national registry selected a matching number of consecutively recruited conservatively managed controls for comparison. As the aim was to investigate the clinical and angiographic differences between PCI and conservative cohorts (rather than to investigate outcomes in patients with equivalent presentations), cases were selected blinded to clinical and angiographic findings and without prior matching for demographic or angiographic features. Any cases or controls not felt to be definite SCAD by any observer during the subsequent angiographic analysis were excluded. This small number of exclusions means the final analysed groups are of slightly different sizes. Iatrogenic dissection cases were only included if there was clear evidence of SCAD preceding and/or anatomically remote from the iatrogenic injury. Iatrogenic dissections were further classified according to whether they occurred during the diagnostic or interventional phase of the procedure.

### Confirmation of SCAD diagnosis

All patients had an angiographically confirmed diagnosis of SCAD on image review. All angiograms, with intracoronary imaging where available, were assessed by experienced interventional cardiologists representing at least two of the national registries with any initial disagreements resolved by consensus. Patients with atherosclerotic, traumatic or iatrogenic dissection (except where the latter complicated definite SCAD) were excluded.

### Patient, SCAD and intervention characteristics

Demographic information, medical history and a detailed history of the SCAD event were obtained from the patient’s medical record. PCI procedure details were collected from procedure reports. Clinical characteristics included: myocardial infarction type (cardiac arrest, non-ST-elevation myocardial infarction (NSTEMI) or ST-elevation myocardial infarction (STEMI)) - STEMI was defined as typical ECG changes occurring at any stage prior to angiography; cardiac arrest as any cessation of circulation requiring resuscitation occurring before the diagnostic angiographic procedure), pregnancy-associated SCAD (P-SCAD) as SCAD occurring during gestation or within 12 months of delivery. Not all registries held the full contemporaneous medical records to allow an accurate determination of admission blood pressure to reliably determine cardiogenic shock according to accepted standard definitions. These data were therefore not included.

### Angiographic analysis

Details of SCAD findings were recorded from angiographic images. Additionally, see online supplemental data.

### Outcomes

PCI complications were recorded from angiographic images and defined as: aorto-ostial iatrogenic dissection; haematoma extension; loss of flow in the stented vessel or a significant side branch (>2 mm diameter); and vessel perforation as shown by extravasation of contrast (contrast penetration of the false lumen during PCI was not considered a complication). Serious PCI complications were defined as aorto-ostial iatrogenic dissection, complications resulting in reduced flow in a proximal epicardial coronary vessel or leading to unplanned left mainstem (LMS) stenting or CABG. There is no established definition of SCAD-PCI complications, although these are acknowledged to be somewhat different from those in atherosclerotic ACS. The interventional representatives of the three national registries therefore adopted these consensus definitions, largely based on those reported in related studies.^1 2^

Postoperative outcomes were major adverse cardiovascular and cerebrovascular events (MACCEs; death, stroke, myocardial infarction or revascularisation occurring at any point after discharge from the index SCAD event) and recurrence (a new angiographically confirmed SCAD occurring after discharge from the index episode and either anatomically or temporally separated from the first event). Time to recurrence and time to MACCE were also recorded.

### Statistical analysis

Patient, clinical and intervention characteristics were summarised by median and IQR for continuous measures or number and percentage for categorical variables. Comparisons of number and total length of stents between SCAD-PCI patients who developed complications and uncomplicated SCAD-PCI patients were made using Kruskal-Wallis test as the variables were not normally distributed.

Logistic regression was used to calculate the OR and 95% CIs of the risk of any complication and serious complications associated with patient, clinical and intervention characteristics. Unadjusted and two adjusted models were created. The first adjusted model included age, sex and ethnicity. The second adjusted model included additionally all patient, clinical and intervention characteristics significantly associated with the outcome of interest. The latter was performed to assess which of the characteristics remained significant when all variables were adjusted for (eg, total stent length and number of stents). Due to the inclusion of variables pertaining to stents, only stented individuals were included in the analyses. An a priori decision was made that interaction terms would not be included, as we did not believe that the joint effect of any two of these variables would be higher than expected from the sum of their individual effects.

Two Kaplan-Meier plots were constructed to compare: (1) time to MACCE and (2) time to recurrence for SCAD-PCI and SCAD-non-PCI patients. As the proportional hazards assumption was met, as assessed by Kaplan-Meier plot and the test of Grambsch-Therneau,^11^ Cox proportional hazards regression models were used to calculate the HR and 95% CI for the risk of MACCE and recurrence associated with patient, clinical and intervention characteristics, with adjustment for age, sex and ethnicity. To assess differences in risk between SCAD-PCI and SCAD-non-PCI patients, an interaction term between clinical management (PCI vs conservative) and the predictor was included.

All statistical analyses were performed using STATA release V.16 (Stata Corp, College Station, Texas, USA) and R for statistical computing.^12^

## Results

### Cohort demographics

Two hundred and fifteen SCAD-PCI patients were included. Patient, clinical and intervention characteristics are summarised in table 1. Patients were predominantly female and white European with a median age at the time of SCAD of 48 years. In the SCAD PCI cohort there were 12 (5.6%) P-SCAD cases.

**Table 1:** Descriptive characteristics of the SCAD cohort, by clinical management

	Conservative (n=221) (n (%))		PCI (n=215) (n (%))	
**Patient characteristics**				
Total	221	50.7	215	49.3
Age at first SCAD event, years (median, IQR)	49	(43–55)	48	(42–54)
Ethnicity*				
White European	212	95.9	197	92.5
Not white European	9	4.1	16	7.5
Sex				
Female	203	91.9	203	94.4
Male	18	8.1	12	5.6
Pregnancy status				
Not pregnant (female)	192	86.9	191	88.8
Pregnant (female)	11	5.0	12	5.6
Smoking				
Never smokers	140	(63.3)	146	(67.9)
Ex-smokers	55	(24.9)	44	(20.5)
Current smokers	26	(11.8)	25	(11.6)
Diabetes mellitus				
No	>216	(98.2)	>210	(99.1)
Yes	<5	(1.8)	<5	(0.9)
Hypertension				
No	162	(73.3)	166	(77.2)
Yes	59	(26.7)	49	(22.8)
Dyslipidaemia				
No	185	(83.7)	187	(87.0)
Yes	36	(16.3)	28	(13.0)
**Clinical characteristics**				
NSTEMI	131	59.3	74	34.4
STEMI	77	34.8	119	55.3
Cardiac arrest	13	5.9	22	10.2
Left main stem vessel affected	<5	2.3	14	6.5
Left anterior descending artery affected	112	50.7	155	72.1
Left circumflex artery affected	83	37.6	48	22.3
Right coronary artery affected	58	26.2	29	13.5
AHA coronary segment involved				
Proximal	17	7.7%	64	29.8%
Mid	52	23.5%	78	36.3%
Distal	84	38.0%	45	20.9%
Branch	68	30.8%	28	13.0%
More than one vessel involved*	32	14.5%	19	8.8%
More than one segment in the vessel involved	60	27.1%	83	38.6%
Tortuosity index* (median, IQR)	4	(2-6)	3	(2-5)
Type 1	28	12.7%	26	12.1%
Type 2	153	69.3%	118	54.9%
Type 3	26	11.8%	14	6.5%
Type 4	14	6.3%	57	26.5%
Taking aspirin*	205	94.5%	210	98.1%
Taking DAPT*	161†	74.5%	193†	91.0%
Taking beta-blocker*	173	79.7%	189	88.3%
Taking ACE inhibitors*	146	67.0%	158	73.8%
Taking statins*	155	71.1%	171	80.3%
**Intervention quantitative coronary analysis**				
Type of intervention				
Conservative	221	100.0%	–	–
Stent	0	0.0%	156	72.6%
Balloon	0	0.0%	45	20.9%
Wiring	0	0.0%	14	6.5%
Maximum stent diameter, mm (median, IQR)	–	–	3.0	(2.5–3.5)
Total number of stents (median, IQR)	–	–	2	(1-3)
Total length of stents, cm (median, IQR)	–	–	46	(28-61)
Proximal diameter, mm (median, IQR)	–	–	2.53	(2.15–3.1)
Length of lesion, mm (median, IQR)	–	–	38.6	(26.6–56.2)
Total volume of haematoma, mm^3^ (median, IQR)	–	–	57.0	(30.0–102.7)
Final TIMI grade flow				
0 (no flow)	–	–	16	7.4%
1	–	–	11	5.1%
2	–	–	23	10.7%
3 (good flow)	–	–	165	76.7%
Outcomes				
Any complication*	–	–	83	38.6%
Serious complication*	–	–	28	13.0%
Time to MACCE (median, IQR)	2.10	(1.06–3.61)	2.32	(1.04–4.28)
MACCE	22	9.5%	31	14.4%
Time to recurrence* (median, IQR)	2.5	(1.2–4.7)	4.4	(2.7–6.3)
Recurrence*	15	6.8%	13	6.1%

*Missing values: ethnicity/race: 2 (0.5%), maximum stent diameter: 74 (34.4%), total number of stents: 1 (0.6%), total stent length: 14 (9.0%), proximal diameter: 47 (21.9%), lesion length: 74 (34.4%), volume of haematoma: 79 (36.7%), tortuosity index: 1 (0.2%), time to recurrence: 2 (0.5%), aspirin: 5 (1.2%), DAPT: 8 (1.8%), beta-blocker: 5 (1.2%), ACE: 4 (0.9%), statin: 5 (1.2%), recurrence: 2 (0.5%).

†Of those not taking DAPT at discharge, three in the PCI group and two in the non-PCI group were anticoagulated. Of those taking DAPT, 65%, 30% and 4% in the non-PCI group and 37%, 49% and 14% in the PCI group were taking clopdiogrel, ticagrelor or prasugrel, respectively.

DAPT, dual antiplatelet therapy; NSTEMI, non-ST-elevation myocardial infarction; PCI, percutaneous coronary intervention; SCAD, spontaneous coronary artery dissection; STEMI, ST-elevation myocardial infarction; TIMI, thrombolysis in myocardial infarction.

### Clinical and angiographic presentation

**Figure 1:**
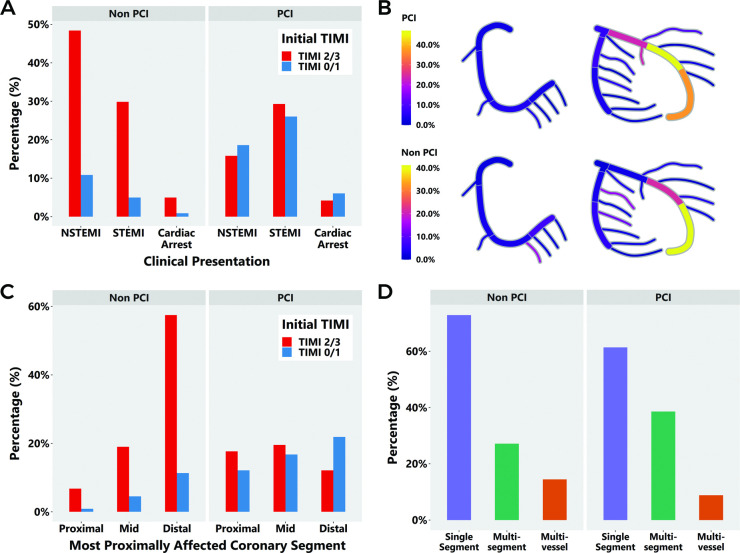
Clinical presentation of SCAD-PCI (n=215) and SCAD-non-PCI patients (n=221), including (A) initial presentation, (B) coronary heatmap of affected segments (see also online supplemental efigure 1 for full segment-by-segment analysis), (C) TIMI flow and (D) multisegment and multivessel disease. PCI, percutaneous coronary intervention; SCAD, spontaneous coronary artery dissection; TIMI, thrombolysis in myocardial infarction.

Two hundred and twenty-one blindly selected SCAD-non-PCI controls were included for comparison of clinical presentation as shown in figure 1 and table 1. SCAD-PCI patients were more likely to present with STEMI and with TIMI 0/1 than SCAD-non-PCI (figure 1A, table 1). In keeping with this, while Yip-Saw classification type 2 SCAD was the most common angiographic class at presentation, SCAD-PCI patients were more likely to present with Yip-Saw classification type 4 (occlusions) compared with SCAD non-PCI patients (table 1, online supplemental efigure 1A).

A radial initial approach was undertaken in 74.3% of SCAD-PCI patients and in 84.2% of SCAD non-PCI patients. The left anterior descending coronary artery was the most affected vessel. A coronary heatmap of affected coronary segments is shown in figure 1B (a graph of all affected American Heart Association (AHA) coronary segments is shown in online supplemental efigure 1B). There was greater involvement of proximal and midvessel lesions in the SCAD-PCI compared with the SCAD-non-PCI group (table 1, figure 1C). There was more multisegment disease but not multivessel disease in SCAD-PCI patients than SCAD-non-PCI patients (table 1, figure 1D).

SCAD-PCI lesion characteristics, as assessed by quantitative coronary angiography (3D-QCA) are shown in online supplemental efigure 2. The median upstream vessel diameter was 2.7 mm (IQR: 2.3–3.2, n=168) with a downstream diameter of 1.8 mm (IQR: 1.5–2.3, n=126) (online supplemental figure 2A). The median minimal luminal diameter was 0.6 mm (IQR: 0.4–0.9, n=124), and the median minimal luminal area was 0.5 mm^2^ (IQR: 0.3–1.1 mm^2^, n=121; online supplemental efigure 2B). The median of the measured lesion bending angles was 47° (IQR: 34–61°, n=148), which was close to the maximum bending angle for affected vessels (median 55°; IQR: 43–70°, n=168) (online supplemental efigure 2C). The median volume of haematoma displaced by stenting was 54.0 mm^3^ (IQR: 27.3–113.9 mm^3^, n=86) (online supplemental efigure 2D).

Nine patients underwent intracoronary imaging only and were included in the SCAD-non-PCI group, 62 patients underwent intracoronary imaging as part of SCAD-PCI.

### Characteristics of SCAD revascularisation

**Figure 2:**
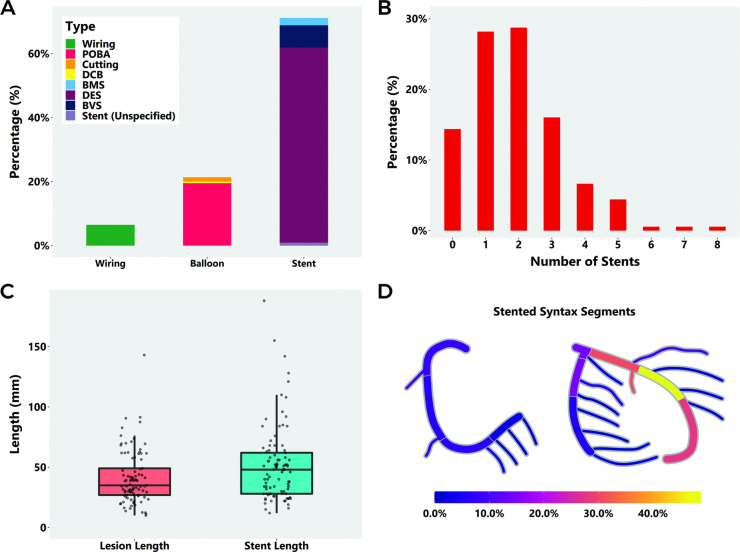
Details of the PCI procedure in SCAD intervention patients (n=215): (A) interventional strategy, (B) number of stents deployed, (C) stent length compared with lesion length and (D) coronary heat map of stented AHA coronary segments. AHA, American Heart Association; PCI, percutaneous coronary intervention; SCAD, spontaneous coronary artery dissection.

Of patients undergoing PCI, 7.0% (15/215) had failed an initial trial of conservative management. Four patients underwent emergency CABG, two as a planned revascularisation strategy after angiography and two after failed or complicated PCI. The nature of the PCI procedure undertaken is shown in figure 2A, 72.6% (156/215) of patients underwent stenting, mostly with drug eluting stents, 20.9% (45/215) had balloon angioplasty, 5 with cutting balloons and 6.5% (14/215) underwent wiring only. The number of stents deployed is shown in figure 2B (mean 2.3 (range 1–8) per stented case). Twenty-three cases (10.6% of all SCAD-PCI cases) required four or more stents. In the 142 patients with both total number of stents and total stent length recorded, the median total length of deployed stents was 46 mm (IQR: 28.5–60.8 mm; mean 51.5 mm) with 64 cases (29.8% of all SCAD-PCI cases) requiring ≥50 mm stents (figure 2C). 64.1% (100/156) of stented cases were left with residual unstented areas of dissection. The median diameter of the smallest deployed stent in each case was 2.5 mm (IQR: 2.5–3.0 mm). Figure 2D shows a coronary heatmap of sites of stenting also shown by AHA coronary segment. Areas of residual dissection mostly occurred in distal coronary locations such as AHA coronary segments, 8, 4, 14 and 16, while initially unaffected segments that were more frequently stented were mainly in proximal segments (1, 5 and 11). 10.3% (16/156) of stented cases required stenting into the LMS.

Of all SCAD-PCI cases, 33.0% (71/215) required one or more of either unplanned LMS stenting, excessive stent number (≥4) or stent length (≥50 mm).

### Outcomes of SCAD-PCI

**Figure 3:**
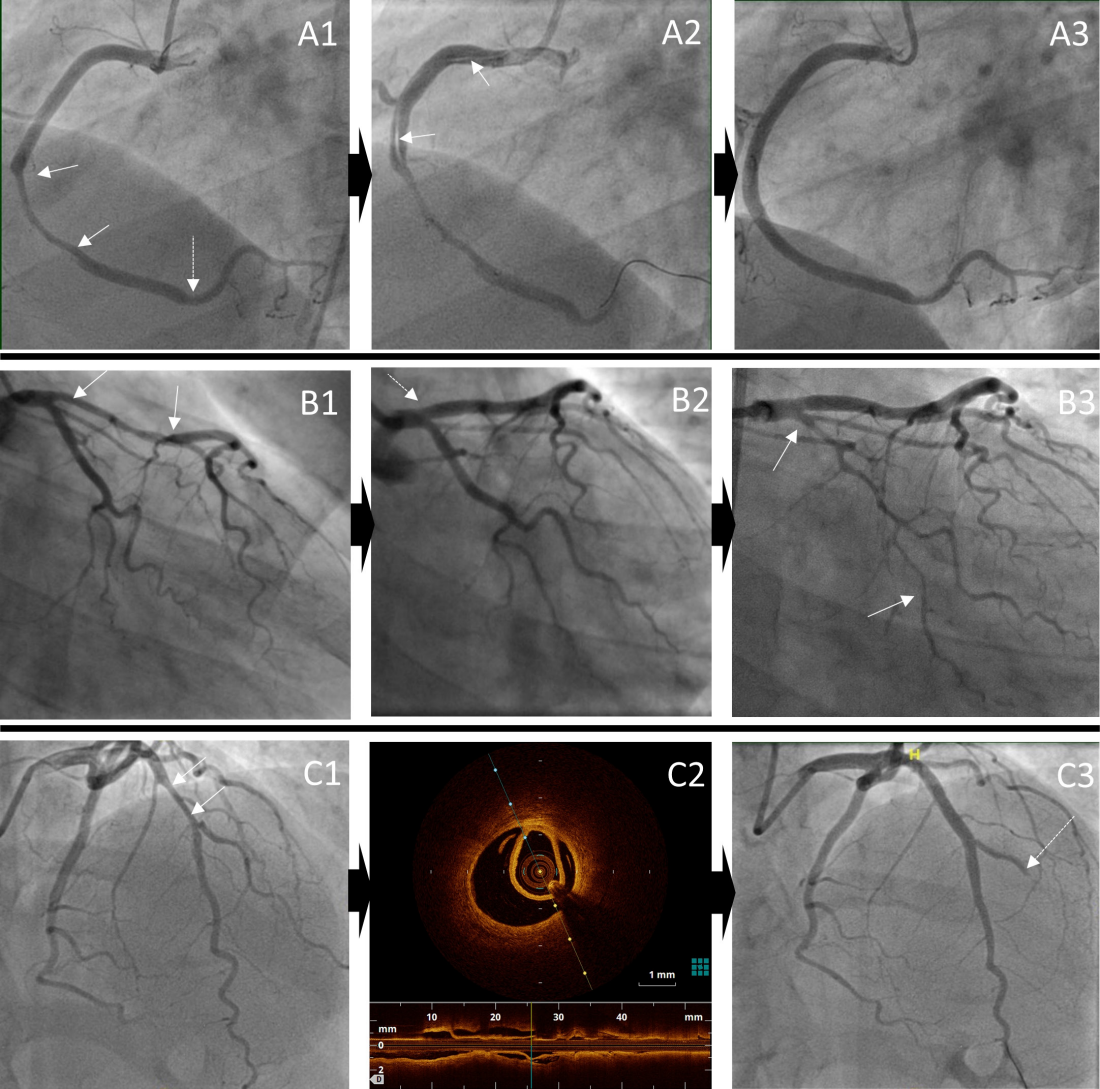
Angiographic exemplars of complications (both serious and not serious) occurring during percutaneous coronary intervention. Iatrogenic dissection: A1: extensive dissection of mid-right coronary artery (solid arrows) with further haematoma distally (dotted arrow), A2: iatrogenic dissection from ostium to mid vessel and A3: extensive stenting from proximal to distal vessel; Haematoma extension: B1: proximal-mid left anterior descending haematoma (solid arrows), B2: treated with stenting to the ostium, initially with only small prestent residual haematoma (dotted arrow), B3: but later leading to propagation of haematoma into circumflex (solid arrows); Side-branch occlusion: C1: mid-left anterior descending stenosis (solid arrows), C2: confirmed on OCT to be due to SCAD and C3: stenting leads to occlusion of diagonal branch (dotted arrow). OCT, optical coherence tomography; SCAD, spontaneous coronary artery dissection.

Angiographic exemplars of typical complications occurring during PCI are shown in figure 3.

**Figure 4:**
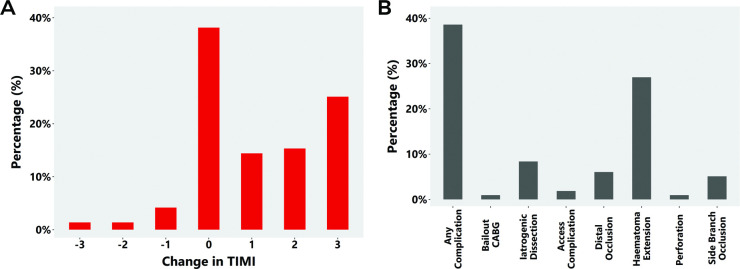
(A) Changes in thrombolysis in myocardial infarction (TIMI) flow for SCAD-PCI patients and (B) PCI complications in the SCAD-PCI cohort (n=215). CABG, coronary artery bypass grafting; PCI, percutaneous coronary intervention; SCAD, spontaneous coronary artery dissection.

The impact of PCI on TIMI flow is shown in figure 4A (with findings for the SCAD-STEMI subpopulation shown in online supplemental efigure 4A). In those with reduced TIMI flow, improvements were seen in 84.3% (118/140) of patients. In all patients undergoing PCI, worsening of TIMI flow was seen in 7.0% (15/215) (figure 4A). Changes in TIMI flow according to initial TIMI are shown in online supplemental efigure 3.

PCI complications occurred in 38.6% (83/215) of SCAD-PCI patients with the the most common being haematoma extension (58/215, 27.0%) and iatrogenic dissection (18/215, 8.4%; nine occurring during the diagnostic phase and nine after initiation of PCI) (figure 4B). Two complications occurred during intracoronary imaging, the remainder during SCAD-PCI. A similar frequency and distribution of complications was noted in the SCAD-STEMI subpopulation (online supplemental efigure 4B). Stenting into the false lumen was noted in one case.

Compared with patients without complications, patients who developed complications had a longer stent length (median 52 (IQR: 38–64) PCI complications; median 35 (IQR: 26.8–56) PCI no complications; p=0.004). Despite this, 47.0% (39/83) showed improvements in TIMI flow grade at procedure end, 12.0% (10/83) showed a deterioration and 41.0% (34/83) no change in TIMI flow grade. Of those with impaired TIMI flow grade at the start of the procedure, 33/45 (73.3%) showed an improvement in flow in at least one AHA coronary segment of a major epicardial coronary territory, while only 5 (11.1%) showed a deterioration. Subjectively in terms of overall angiographic coronary perfusion, 44 of 83 (53.0%) cases with complications showed an improved situation at procedure end, 17 (20.5%) showed a deterioration and 22 (26.5%) showed no overall change.

13.0 (28/215) of SCAD patients undergoing PCI suffered subjectively serious complications (16 cases of iatrogenic dissection, 16 cases requiring unplanned LMS stenting, 3 cases demonstrated loss of flow in a proximal AHA coronary segment and 2 patients required CABG because of PCI failure).

Predictors of any PCI complications and serious complications are shown in table 2 and online supplemental etable 1, respectively. Predictors of complications grouped into markers of more extensive dissections (lesion length, volume of haematoma and multisegment dissections), more proximal dissections (proximal vs distal, proximal diameter and maximum stent diameter) and a lack of angiographic contrast penetration of the false lumen (Yip-Saw classification type 2). Of these multisegment dissections, LMS dissections and the use of larger diameter stents remained predictors of serious complications.

**Table 2:** Association between patient, clinical and intervention characteristics and odds of any complication in a SCAD cohort

	Unadjusted			Age, sex and ethnicity adjusted		
	Cases/n	OR (95% CI)	P value	Cases/n	OR (95% CI)	P value
**Patient characteristics**						
Age at first SCAD event, per year	83/215	1.01 (0.98 to 1.04)	0.631	n/a	n/a	n/a
Ethnicity (white European vs non-white European)*	83/213	0.81 (0.29 to 2.26)	0.684	n/a	n/a	n/a
Male versus female	83/215	0.78 (0.23 to 2.69)	0.658	n/a	n/a	n/a
Pregnant female versus non-pregnant female	79/203	0.50 (0.13 to 1.92)	0.316	79/201	0.51 (0.12 to 2.15)	0.360
Grading of tortuosity for all vessels imaged, per unit*	83/214	0.95 (0.85 to 1.07)	0.437	83/213	0.94 (0.83 to 1.06)	0.349
**Clinical characteristics**						
Type of myocardial infarction						
STEMI versus NSTEMI	83/215	0.92 (0.51 to 1.67)	0.794	83/213	0.91 (0.50 to 1.65)	0.753
Cardiac arrest versus NSTEMI		0.68 (0.25 to 1.88)	0.462		0.74 (0.27 to 2.08)	0.573
Left main stem vessel affected	83/215	1.64 (0.56 to 4.87)	0.369	83/217	1.67 (0.55 to 5.12)	0.366
Left anterior descending artery affected	83/215	0.92 (0.50 to 1.70)	0.794	83/217	0.90 (0.49 to 1.66)	0.736
Left circumflex artery affected	83/215	1.05 (0.55 to 2.03)	0.874	83/217	1.05 (0.55 to 2.02)	0.879
Right coronary artery affected	83/215	1.14 (0.52 to 2.53)	0.742	83/217	1.18 (0.54 to 2.59)	0.679
AHA coronary segment involved						
Mid versus proximal	83/215	0.79 (0.40 to 1.54)	0.485	83/213	0.74 (0.37 to 1.46)	0.383
Distal versus proximal		0.32 (0.14 to 0.76)	0.010		0.29 (0.12 to 0.71)	0.007
Branch versus proximal		0.73 (0.30 to 1.81)	0.501		0.65 (0.26 to 1.66)	0.367
More than one vessel involved	83/215	0.92 (0.35 to 2.44)	0.869	83/213	1.03 (0.38 to 2.78)	0.960
More than one segment within the vessel involved	83/215	1.77 (1.01 to 3.11)	0.046	83/213	1.90 (1.06 to 3.39)	0.030
Yip-Saw classification based on appearance when first imaged						
Type 2 versus type 1	83/215	2.90 (1.13 to 7.42)	0.026	83/213	2.89 (1.12 to 7.43)	0.028
Type 3 versus type 1		2.71 (0.70 to 10.57)	0.150		2.70 (0.69 to 10.64)	0.155
Type 4 versus type 1		0.44 (0.14 to 1.39)	0.163		0.42 (0.13 to 1.33)	0.141
**Intervention details**						
Type of intervention						
Balloon versus stent	83/215	0.08 (0.02 to 0.26)	<0.001	83/213	0.07 (0.02 to 0.24)	<0.001
Wiring versus stent		0.60 (0.19 to 1.87)	0.379		0.53 (0.17 to 1.68)	0.278
Maximum stent diameter, per mm*	65/141	1.83 (1.04 to 3.25)	0.037	65/140	1.93 (1.06 to 3.49)	0.030
Total number of stents, per additional stent*	74/155	1.39 (1.06 to 1.80)	0.015	74/154	1.48 (1.12 to 1.95)	0.006
Total length of stents, per mm*	66/142	1.01 (1.00 to 1.02)	0.137	66/141	1.01 (1.00 to 1.02)	0.049
Proximal diameter, per mm*	65/168	2.11 (1.32 to 3.37)	0.002	65/166	2.25 (1.38 to 3.67)	0.001
Length of lesion, per mm*	59/141	1.02 (1.00 to 1.03)	0.068	59/139	1.02 (1.00 to 1.03)	0.060
Volume of haematoma, per mm^3^*	59/136	1.01 (1.00 to 1.01)	0.016	59/135	1.01 (1.00 to 1.01)	0.014
TIMI flow						
1 versus 0 (no flow)	83/215	0.65 (0.14 to 3.04)	0.582	83/213	0.66 (0.14 to 3.10)	0.596
2 versus 0 (no flow)		0.85 (0.24 to 3.06)	0.802		0.86 (0.24 to 3.12)	0.817
3 (good flow) versus 0 (no flow)		0.41 (0.15 to 1.16)	0.093		0.42 (0.15 to 1.21)	0.107

Only individuals who underwent stenting were included in analyses of the maximum of stent diameter, the total number of stents and the total stent length.

ARB, angiotensin II receptor blockers; DAPT, dual antiplatelet therapy; NSTEMI, non-ST-elevation myocardial infarction; SCAD, spontaneous coronary artery dissection; STEMI, ST-elevation myocardial infarction; TIMI, thrombolysis in myocardial infarction.

When all variables associated with the outcome were included in the model, along with age, sex and ethnicity, the total number of stents remained associated with the risk of any complication in SCAD-PCI patients (OR=1.90, 95% CI 1.26 to 2.85), and the maximum stent diameter remained associated with risk of serious complications in SCAD-PCI patients (OR=2.62, 95% CI 1.28 to 5.39) (online supplemental etable 2 and 3).

### Long-term outcomes

Follow-up information was available on 436 patients (median follow-up 900 days, IQR: 440–1590). Most patients retained good cardiac ejection fraction at follow-up regardless of revascularisation strategy (online supplemental efigure 5). There was no difference in MACCE events between SCAD-PCI and SCAD-non-PCI patients (online supplemental efigure 6A, PCI: 14.4%, 31/215, non-PCI: 9.5%, 21/221; MACCE components - recurrent AMI (PCI: 9.3%, 20/215, non-PCI: 7.7%, 17/221); revascularisation (PCI: 4.7%, non-PCI: 1.4%); stroke (PCI: 1.5%, non-PCI: 0.7%); death (PCI: 1.4%, non-PCI: 0.5%)). There was also no difference in the incidence of recurrent SCAD between the PCI and non-PCI groups (online supplemental efigure 6B, PCI: 6.1%, 13/214, non-PCI: 6.8%, 15/220).

## Discussion

We present the largest international observational study of PCI in SCAD. We report first that PCI in current SCAD practice is appropriately reserved for more serious clinical and angiographic presentations including STEMI, reduced TIMI flow and proximal disease. Second, that complications in SCAD-PCI are high, occurring in 38.6% of cases, one-third of which are serious (defined as iatrogenic dissection, unplanned LMS stenting, loss of flow in proximal AHA coronary segment and emergency CABG). Third, that despite the higher rates of complications and longer stent lengths required for SCAD-PCI, intervention in this context generally resulted in improvements in measures of coronary flow. Finally, that measures of more extensive and more proximal dissections as well as those without contrast penetration into the false lumen are associated with a higher risk of complications.

The first important risk of PCI in SCAD is the requirement for multiple, often small calibre stents and longer stent lengths. SCAD-PCI on average required an extra 0.7 stents and an extra 21.4 mm of stent compared with UK national audit data (mean 1.64 stents; 30.1 mm stented length per stented case), where atherosclerotic disease will predominate.^13^ Overall, one-third of SCAD-PCI patients required at least one of unplanned LMS PCI, ≥4 stents or ≥50 mm stent length. Furthermore, despite extensive stenting, there remained unstented dissection in 64.1% of SCAD-PCI cases. Despite this, over the duration of available MACCE follow-up (median 30 months), there was no overall increase in MACCE in the SCAD-PCI group (despite the more severe characteristics at presentation). Importantly, longer term adverse sequelae of the more extensive stenting in this relatively young SCAD population are currently unclear, and further data are needed to assess this.

The high rate of complications is another risk of SCAD-PCI, which has been widely reported^4 5^ and is confirmed in this study. However, in keeping with a previous study in SCAD-STEMI,^14^ most patients undergoing PCI in this study were high risk at presentation with only 8.8% undergoing PCI outside the context of STEMI/cardiac arrest, TIMI 0/1 flow or proximal dissections. Conservative management in these patients would likely incur higher jeopardy. Furthermore, this analysis confirms that, even within this group, complications are more likely to occur in patients with more extensive and more proximal dissections, where larger volumes of false lumen haematoma pose a greater risk from displacement or propagation during stenting. Also of potential clinical utility is the finding that angiographic features of intramural haematoma without contrast penetration of the false lumen (Yip-Saw classification type 2) were more associated with complications, suggesting perhaps that connections between true and false lumen allow decompression of the false lumen with stent inflation, making haematoma propagation and therefore PCI complications less likely. Stent number and stent length also correlated with PCI complications, but for these factors, it is difficult to determine cause versus effect. Coronary tortuosity, known to be increased in SCAD,^15^ did not increase the risk of PCI complications.

There were also important benefits of SCAD-PCI. Improvements in TIMI flow grade were seen after PCI in 84.3% of cases with 38.6% improving three grades, 23.6% improving two grades and 22.1% improving one grade. Only 7.0% of cases saw a deterioration in TIMI flow grade with PCI. Interestingly, these gains were mostly seen in cases where stenting rather than more limited wiring or plain old balloon angioplasty strategies was adopted.

Taken together these data suggest that PCI should remain the reserve of high-risk SCAD presentations. Operators should be aware that the risk of complications is higher than for atherosclerotic cases and that long lengths of stent may be required. Cases with extensive dissections, proximal dissections and those with no contrast penetration into the false lumen may be at particularly high risk. However, despite this, it is possible in most of these high-risk cases to achieve subjective improvements in coronary perfusion. It should be noted that there is also potential to further improve the risk–benefit balance in SCAD-PCI by considering measures to reduce potentially avoidable complications such as the use of less aggressive more coaxial catheter shapes that might reduce the risk of iatrogenic dissection in patients with clinically possible SCAD. The point of equipoise between PCI and a conservative approach to revascularisation remains unclear. A previous propensity matched analysis of angiographically and clinically matched cases showed no difference in convalescent infarct size between PCI and conservatively managed cases.^16^ This analysis was inevitably largely limited to intermediate risk anatomies. A clinical trial in predefined higher risk anatomies is required to address this question definitively. It was also not possible from this analysis to assess the efficacy of different PCI techniques. Future studies should be designed to investigate this.

### Limitations

This is an observational study, and therefore, we cannot conclude that the associations demonstrated are causative. Our data are derived from a SCAD survivor cohort and do not include information on early SCAD-PCI non-survivors. Although the three national SCAD registries recruit all comers and include patients referred from hospital clinicians, primary care physicians and self-referrals, there remains the potential for selection bias. The non-PCI cohort were selected blinded to clinical and angiographic findings. The difference in cohort sizes is due to a small number of exclusions arising after case selection. Exclusions arose when a case was felt by any observer not to be definite SCAD on review of angiography (plus intracoronary imaging when present) during the analysis phase. Criteria for both complications and serious complications were selected by experienced SCAD interventional cardiologists but are not established definitions. Benchmarking data from the BCIS audit are discussed to allow a meaningful comparison of stenting data. These data are from a single national jurisdiction, although no significant transnational differences were observed online supplemental etable 4. Data on cardiogenic shock were not available, although the reported incidence in larger SCAD series is low (2%^17^).

## Conclusions

While a conservative approach to revascularisation in SCAD is optimal where possible, sometimes the clinical presentation (STEMI, cardiac arrest, poor TIMI flow and proximal occlusive dissection) mandates intervention to improve coronary perfusion and reduce myocardial injury. This study demonstrates that although more extensive stenting may be required, with an elevated risk of procedural complications, improved coronary flow and good medium-term outcomes can be achieved with PCI. More extensive dissection, proximal segment location and an absence of contrast penetration of the false lumen associate with the highest risk of PCI complications.

**Key messages**

**What is already known on this subject?**

- Percutaneous coronary intervention (PCI) in spontaneous coronary artery dissection (SCAD) is associated with high rates of procedural complications.
- A conservative approach to revascularisation is recommended where possible.

**What might this study add?**

- The ‘cost’ of PCI in SCAD is longer stent lengths and more procedural complications.
- The ‘benefit’ of PCI in SCAD is improved coronary flow.
- Outcomes from SCAD-PCI in terms of left ventricular function and major adverse cardiovascular and cerebrovascular events are generally good.

**How might this impact on clinical practice?**

- Case selection for PCI in SCAD is critical. Most cases can be managed conservatively but for high-risk cases (eg, proximal to mid vessel occlusions) intervention is required.
- While PCI is associated with a significant risk of procedural complications requiring long stent lengths, improved flow and good long-term outcomes can be achieved.

## Data Availability

Data from this study are available on reasonable request.
