# Supplementary material for: Risks and benefits of percutaneous coronary intervention in spontaneous coronary artery dissection
Source: Heart. 2021 May 18;107(17):1398–406. doi: 10.1136/heartjnl-2020-318914 (PMC8372386; doi:10.1136/heartjnl-2020-318914)
Supplement: Supplementary data [file heartjnl-2020-318914supp001.pdf]

Supplemental Material

Risks and benefits of Percutaneous Coronary Intervention in Spontaneous Coronary Artery Dissection

Deevia Kotecha MBBS[1]*, Marcos Garcia-Guimaraes MD[1,2, 3]*, Diluka Premawardhana MBBS[1]*, Dario Pellegrini MD[4, 5], Clare Oliver-Williams PhD[1], Vasiliki Boutziouka PhD[1], Alice Wood MBBS[1], Nalin Natarajan MBBS[1], Robert Jackson MBBS[1], Nathan Chan MBBS[1], Jan Ziaullah MBBS[1], Roby Rakhit MD[6], Stephen P Hoole MD[7], Thomas W Johnson PhD[8], Jacek Kadziela MD[9], Peter Ludman MD[10], Nilesh J. Samani MD[1], Angela H.E.M. Maas MD[4], Robert Jan Van Geuns MD[4]**, Fernando Alfonso MD[2]** and David Adlam DPhil[1]**

*Joint first authors, **Joint senior authors

[1] Department of Cardiovascular Sciences, and NIHR Leicester Biomedical Research Centre, Leicester, UK

[2] Department of Cardiology, Hospital Universitario de La Princesa, Madrid, Spain

[3] Department of Cardiology, Hospital del Mar, Barcelona, Spain

[4] Radboud University Medical Center, Department of Cardiology, Nijmegen, The Netherlands

[5] ASST Papa Giovanni XXIII, Bergamo, Italy

[6] Royal Free Hospital, London, UK

[7] Royal Papworth Hospital and NIHR Cambridge Biomedical Research Centre, Cambridge, UK

[8] Bristol Heart Institute, University Hospitals Bristol & Weston NHS Foundation Trust, Bristol, UK

[9] Department of Interventional Cardiology and Angiology, National Institute of Cardiology, Warsaw, Poland

[10] Institute of Cardiovascular Sciences, University of Birmingham, UK

Kotecha D, *et al. Heart* 2021; 107:1398–1406. doi: 10.1136/heartjnl-2020-318914

SUPPLEMENTARY METHODS

The UK, Dutch and Spanish SCAD registries

The UK, Dutch and Spanish SCAD registries are national observational registries which have been previously described[1-3]. Each was established independently but under the leadership of the European Society of Cardiology Acute Cardiovascular Care Association SCAD Study Group the registries have been working together to increase the power of observational analyses which can be substantially enhanced in this way. All registries accept referrals of SCAD patients from clinicians and additionally patients can self-refer to the UK registry via a web recruitment portal. As these are consented registries, all patients included are inherently survivors of SCAD with most patients consented after hospital discharge. The registries collect parallel data on patient demographics, past medical history and details of the SCAD presentation. Imaging data collected include the index angiogram and imaging conducted to assess for extra-coronary arteriopathies and at the time of recurrent AMI.

Angiographic analysis

Angiographic analysis was conducted using Medis® Suite software with 3-dimensional quantitative angiography undertaken using Q Angio XA 3D software. Initial analysis was conducted to assess the vessel location, AHA coronary segments [4] and a modified Yip-Saw classification for the SCAD location**.** [5] Multi-vessel disease was defined as discontiguous SCAD locations affecting more than one coronary territory. Multi-segment disease was defined as a single dissection site affecting more than one AHA coronary segment. Proximal disease was defined as dissections involving at least one of AHA coronary segments 1, 5, 6, or 11. 3D QCA analysis was restricted to cases where valid measurement could be made.

Patients with TIMI 0 flow beyond the lesion were excluded. Stent displaced haematoma volume was estimated from the Q Angio XA 3D QCA function [6] which enables estimation of atherosclerotic plaque volume (here interpreted as haematoma volume) by subtracting measured lumen volume from a predicted 'normal' lumen volume based on projected vessel boundaries from the 3D vessel reconstruction. The estimated stent displaced volume was then calculated as:

$$Displaced\ haematoma\ volume = \frac{(Haematoma\ volume\ \times\ Stent\ Length)}{Lesion\ Length}$$

. **References**

1. Al-Hussaini A, Abdelaty A, Gulsin GS, Arnold JR, Garcia-Guimaraes M, Premawardhana D, Budgeon C, Wood A, Natarajan N, Mangion K, Rakhit R, Hoole SP, Johnson TW, Berry C, Hudson I, Gershlick AH, Ladwiniec A, Kovac J, Squire I, Samani NJ, Plein S, McCann GP and Adlam D. Chronic infarct size after spontaneous coronary artery dissection: implications for pathophysiology and clinical management. *Eur Heart J*. 2020.
2. Diez-Villanueva P, Garcia-Guimaraes MM, Macaya F, Masotti M, Nogales JM, Jimenez-Kockar M, Velazquez M, Lozano I, Moreu J, Avanzas P, Salamanca J and Alfonso F. Spontaneous Coronary Artery Dissection and Menopause. *Am J Cardiol*. 2021.
3. Smaardijk VR, Mommersteeg PMC, Kop WJ, Pellegrini D, van Geuns RJ and Maas A. Psychological and clinical characteristics of patients with spontaneous coronary artery dissection: A case-control study. *International journal of cardiology*. 2021;323:1-6.
4. Austen WG, Edwards JE, Frye RL, Gensini GG, Gott VL, Griffith LS, McGoon DC, Murphy ML and Roe BB. A reporting system on patients evaluated for coronary artery disease. Report of the Ad Hoc Committee for Grading of Coronary Artery Disease, Council on Cardiovascular Surgery, American Heart Association. *Circulation*. 1975;51:5-40.
5. Yip A and Saw J. Spontaneous coronary artery dissection-A review. *Cardiovascular diagnosis and therapy*. 2015;5:37-48.
6. Kishi S, Magalhaes TA, Cerci RJ, Zimmermann E, Matheson MB, Vavere A, Tanami Y, Kitslaar PH, George RT, Brinker J, Miller JM, Clouse ME, Lemos PA, Niinuma H, Reiber JHC, Kofoed KF, Rochitte CE, Rybicki FJ, Di Carli MF, Cox C, Lima JAC and Arbab-Zadeh A. Comparative effectiveness of coronary artery stenosis and atherosclerotic plaque burden assessment for predicting 30-day revascularization and 2-year major adverse cardiac events. *Int J Cardiovasc Imaging*. 2020.

Kotecha D, *et al. Heart* 2021; 107:1398–1406. doi: 10.1136/heartjnl-2020-318914

eTable 1: Association between patient, clinical and intervention characteristics and risk of serious complications in a SCAD cohort

		Unadjusted			Age, sex and ethnicity adjusted*		
		n	OR (95% CI)	P	n	OR (95% CI)	P
**Patient Characteristics**							
Age at first SCAD event, per year		215	0.98 (0.94; 1.03)	0.474	n/a	n/a	n/a
Ethnicity (White European vs non-White European)		213	0.63 (0.17; 2.37)	0.494	n/a	n/a	n/a
Male vs Female		215	0.59 (0.07; 4.78)	0.623	n/a	n/a	n/a
Pregnant female vs Non-pregnant female		203	1.33 (0.27; 6.42)	0.724	201	1.08 (0.19; 6.12)	0.933
Grading of tortuosity for all vessels imaged, per unit		214	0.96 (0.81; 1.15)	0.698	213	0.96 (0.80; 1.15)	0.676
**Clinical Characteristics**							
Type of myocardial infarction	STEMI vs NSTEMI	215	0.83 (0.36; 1.91)	0.655	213	0.80 (0.34; 1.85)	0.599
	Cardiac arrest vs NSTEMI		0.57 (0.12; 2.80)	0.492		0.57 (0.12; 2.87)	0.499
Left main stem vessel affected		215	2.95 (0.86; 10.15)	0.086	213	2.84 (0.78; 10.36)	0.114
Left anterior descending artery affected		215	0.55 (0.24; 1.25)	0.154	213	0.49 (0.21; 1.16)	0.106
Left circumflex artery affected		215	1.80 (0.75; 4.28)	0.185	213	1.88 (0.78; 4.53)	0.158
Right coronary artery affected		215	1.94 (0.71;5.30)	0.194	213	2.02 (0.73; 5.61)	0.176
	Mid vs proximal	215	0.53 (0.22; 1.28)	0.156	213	0.52 (0.21; 1.30)	0.160

Kotecha D, *et al. Heart* 2021; 107:1398–1406. doi: 10.1136/heartjnl-2020-318914

AHA coronary segment involved	Distal vs proximal		-	-		-	-
	Branch vs proximal		0.60 (0.18; 2.00)	0.402		0.59 (0.17; 2.09)	0.415
More than one vessel involved		215	2.68 (0.89; 8.15)	0.081	213	2.84 (0.92; 8.80)	0.070
More than one segment within the vessel involved		215	2.38 (1.07; 5.35)	0.034	213	2.38 (1.04; 5.42)	0.038
Yip-Saw Classification based on appearance when imaged	Type 2 vs Type 1	215	1.66 (0.46; 6.04)	0.442	213	1.66 (0.45; 6.07)	0.447
	Type 3 vs Type 1		2.09 (0.36; 12.08)	0.410		1.93 (0.33; 11.27)	0.463
	Type 4 vs Type 1		0.14 (0.01; 1.39)	0.092		0.14 (0.01; 1.40)	0.093
**Intervention Details**							
Type of intervention	Balloon vs stent	215	0.11 (0.01; 0.86)	0.035	213	0.11 (0.01; 0.86)	0.035
	Wiring vs stent		0.38 (0.05; 3.07)	0.367		0.38 (0.05; 3.03)	0.358
Maximum stent diameter, per mm		141	5.40 (2.34; 12.50)	<0.001	140	5.42 (2.29; 12.79)	<0.001
Total number of stents, per additional stent		155	1.63 (1.21; 2.21)	0.002	154	1.68 (1.22; 2.30)	0.001
Total length of stents, per mm		142	1.02 (1.00; 1.03)	0.020	141	1.02 (1.00; 1.03)	0.014
Proximal diameter, per mm		168	0.96 (0.49; 1.88)	0.898	166	0.94 (0.46; 1.89)	0.853
Length of lesion, per mm		141	1.01 (0.99; 1.03)	0.488	139	1.01 (0.99; 1.03)	0.487
Volume of haematoma, per mm^3		136	1.00 (0.99; 1.01)	0.644	135	1.00 (0.99; 1.01)	0.645
TIMI flow	1 vs 0 (No flow)	215	0.96 (0.13; 6.98)	0.970	213	0.92 (0.12; 6.74)	0.933

Kotecha D, *et al. Heart* 2021; 107:1398–1406. doi: 10.1136/heartjnl-2020-318914

2 vs 0 (No flow)	1.90 (0.41; 8.82)	0.415	1.75 (0.37-8.27)	0.478
3 (Good flow) vs 0 (No flow)	0.47 (0.12; 1.81)	0.269	0.44 (0.11; 1.73)	0.237

* Each patient characteristic, clinical characteristic and intervention factor was included separately in a model adjusting for age, sex and ethnicity

ACE - Angiotensin-converting enzyme; ARB - Angiotensin II Receptor Blockers; DAPT – dual antiplatelet therapy; NSTEMI – Non-ST-Elevation Myocardial Infarction; SCAD - Spontaneous Coronary Artery Dissection; STEMI - ST-Elevation Myocardial Infarction; TIMI - Thrombolysis in myocardial infarction

Kotecha D, *et al. Heart* 2021; 107:1398–1406. doi: 10.1136/heartjnl-2020-318914

eTable 2: Risk of any complication with complete data on all predictors in SCAD-PCI patients (n=106)

	Model 1 *		Model 2 *	
	OR (95% CI)	P	OR (95% CI)	P
Age at first SCAD event, per year	-		1.05 (0.99; 1.11)	0.143
Male vs Female	-		0.52 (0.06; 4.56)	0.559
White European vs Not White European	-		1.03 (0.17; 6.17)	0.972
AHA coronary segment involved				
Mid vs proximal	0.83 (0.32; 2.14)	0.696	0.97 (0.29; 3.18)	0.955
Distal vs proximal	0.70 (0.22; 2.28)	0.550	1.64 (0.33; 8.02)	0.544
Branch vs proximal	0.61 (0.17; 2.24)	0.460	1.89 (0.34; 10.42)	0.464
More than one segment within the vessel involved	2.38 (1.06; 5.34)	0.036	1.62 (0.54; 4.86)	0.392
Classification based on appearance when imaged				
Type 2 vs Type 1	4.17 (1.03; 16.83)	0.045	4.21 (0.88; 20.09)	0.072
Type 3 vs Type 1	1.41 (0.16; 12.13)	0.753	1.99 (0.16; 24.46)	0.590
Type 4 vs Type 1	1.23 (0.23; 6.59)	0.813	1.92 (0.29; 12.95)	0.502
Maximum stent diameter, per cm	2.62 (1.28; 5.39)	0.009	1.91 (0.75; 4.86)	0.177
Total number of stents, per additional stent	1.90 (1.26; 2.85)	0.002	1.62 (1.03; 2.56)	0.036
Proximal diameter, per cm	1.93 (1.09; 3.42)	0.031	1.71 (0.84;3.49)	0.142

Model 1: adjusted for age, sex and ethnicity.
Model 2: also adjusted for variables significantly associated with the risk of any complication in Table 2 (AHA coronary segment involved, Classification based on appearance when imaged, maximum stent diameter, total number of stents, proximal diameter)

Intervention type was excluded as this cohort comprised individuals with stents due to the inclusion of maximum stent diameter and total number of stents

SCAD – Spontaneous coronary artery dissection

eTable 3: Risk of serious complications with complete data on all predictors in SCAD-PCI patients (n=140)

	Model 1 *		Model 2 *	
	OR (95% CI)	P	OR (95% CI)	P
Age at first SCAD event, per year	-		1.03 (0.97; 1.10)	0.415
Male vs Female	-		0.53 (0.04; 6.75)	0.623
White European vs Not White European	-		0.28 (0.05; 1.52)	0.140
Left main stem vessel affected	7.24 (1.56; 33.66)	0.012	1.81 (0.29; 11.23)	0.523
More than one segment within the vessel involved	2.35 (0.88;6.30)	0.088	1.36 (0.44; 4.18)	0.7587
Maximum stent diameter, per mm	5.42 (2.29; 12.79)	<0.001	4.41 (1.69; 11.46)	0.002
Total number of stents, per additional stent	1.48 (1.04; 2.11)	0.029	0.77 (0.35;1.73)	0.534
Total length of stents, per mm	1.02 (1.00; 1.03)	0.014	1.02 (0.99; 1.05)	0.315

Model 1: adjusted for age, sex and ethnicity.

Model 2 also adjusted for variables significantly associated with the risk of any complication in Table 2

Kotecha D, *et al. Heart* 2021; 107:1398–1406. doi: 10.1136/heartjnl-2020-318914

eFigure 1: A) Modified Yip-Saw classification type by clinical management, B) AHA coronary segment lesion by clinical management (n=436)

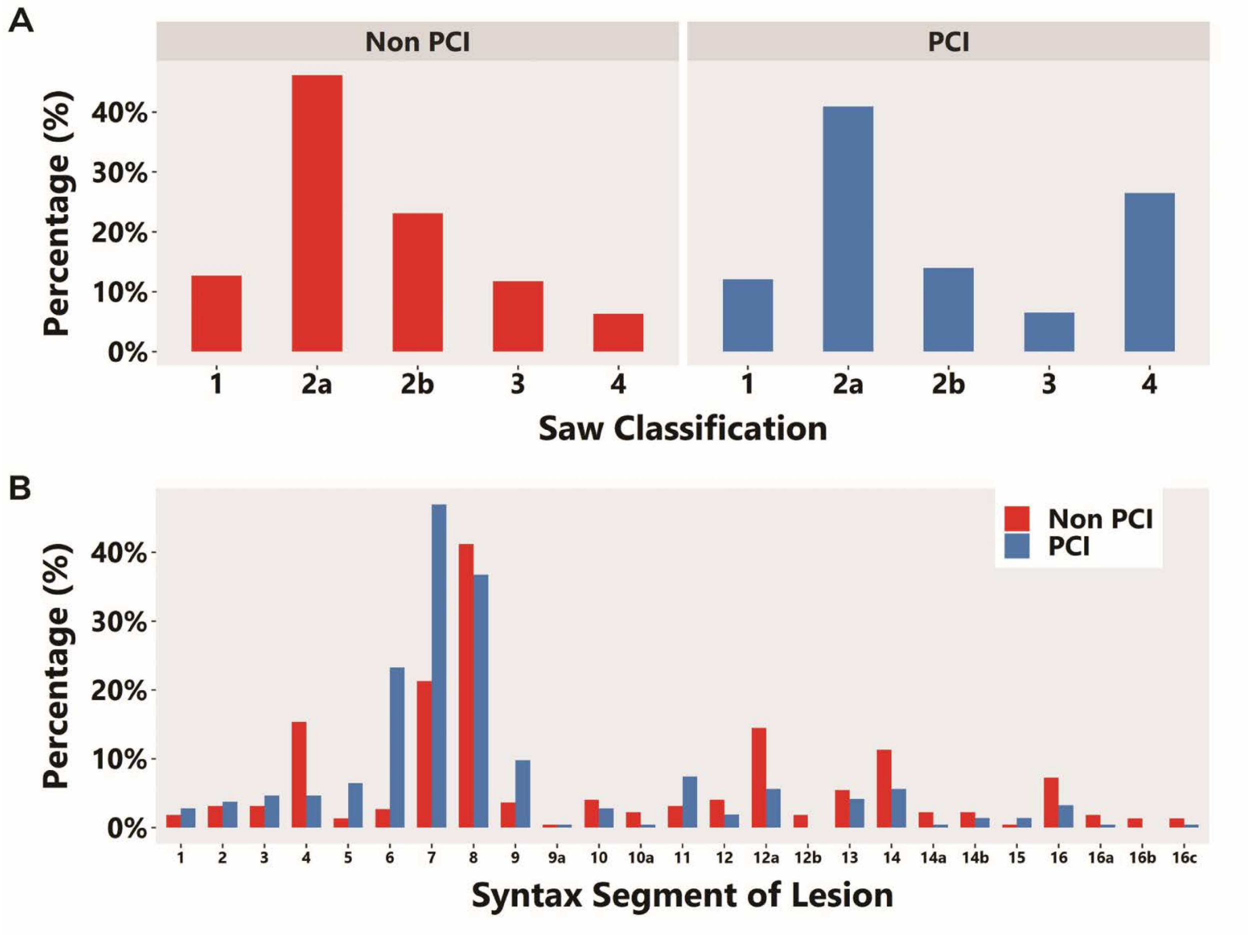

Kotecha D, *et al. Heart* 2021; 107:1398–1406. doi: 10.1136/heartjnl-2020-318914

eFigure 2: SCAD-PCI lesion characteristics as assessed by 3D-QCA (n=215), including A) length, B) area and diameter of stenoses, C) maximum lesion and vessel bending, D) displaced haematoma volume.

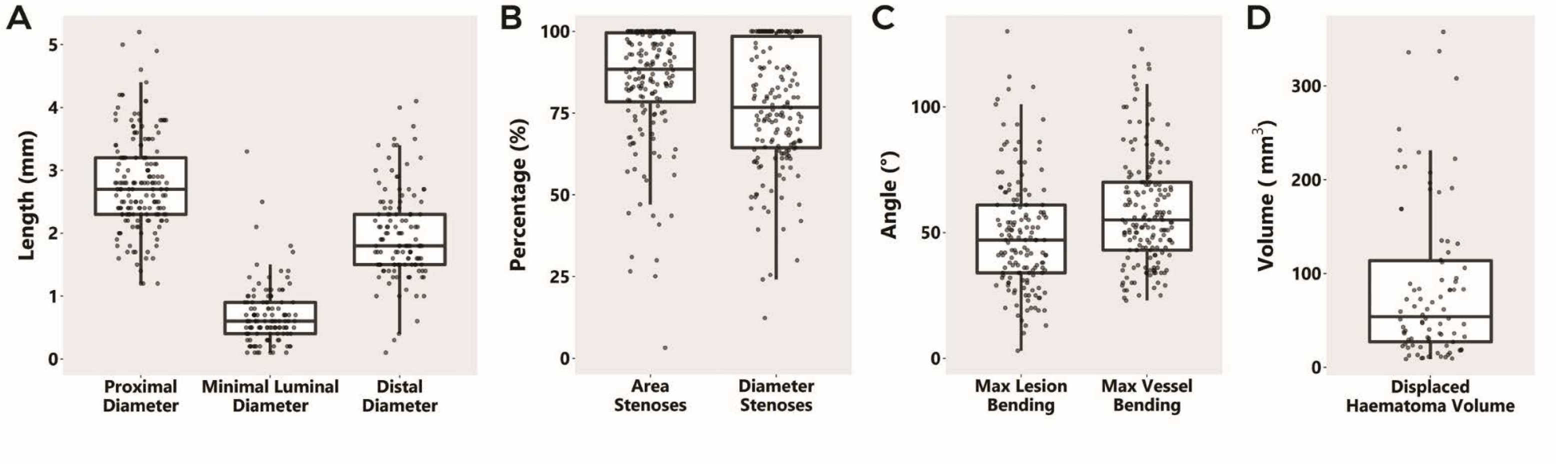

eFigure 3: Change in TIMI grade flow by initial TIMI value in a SCAD-PCI cohort (n=215)

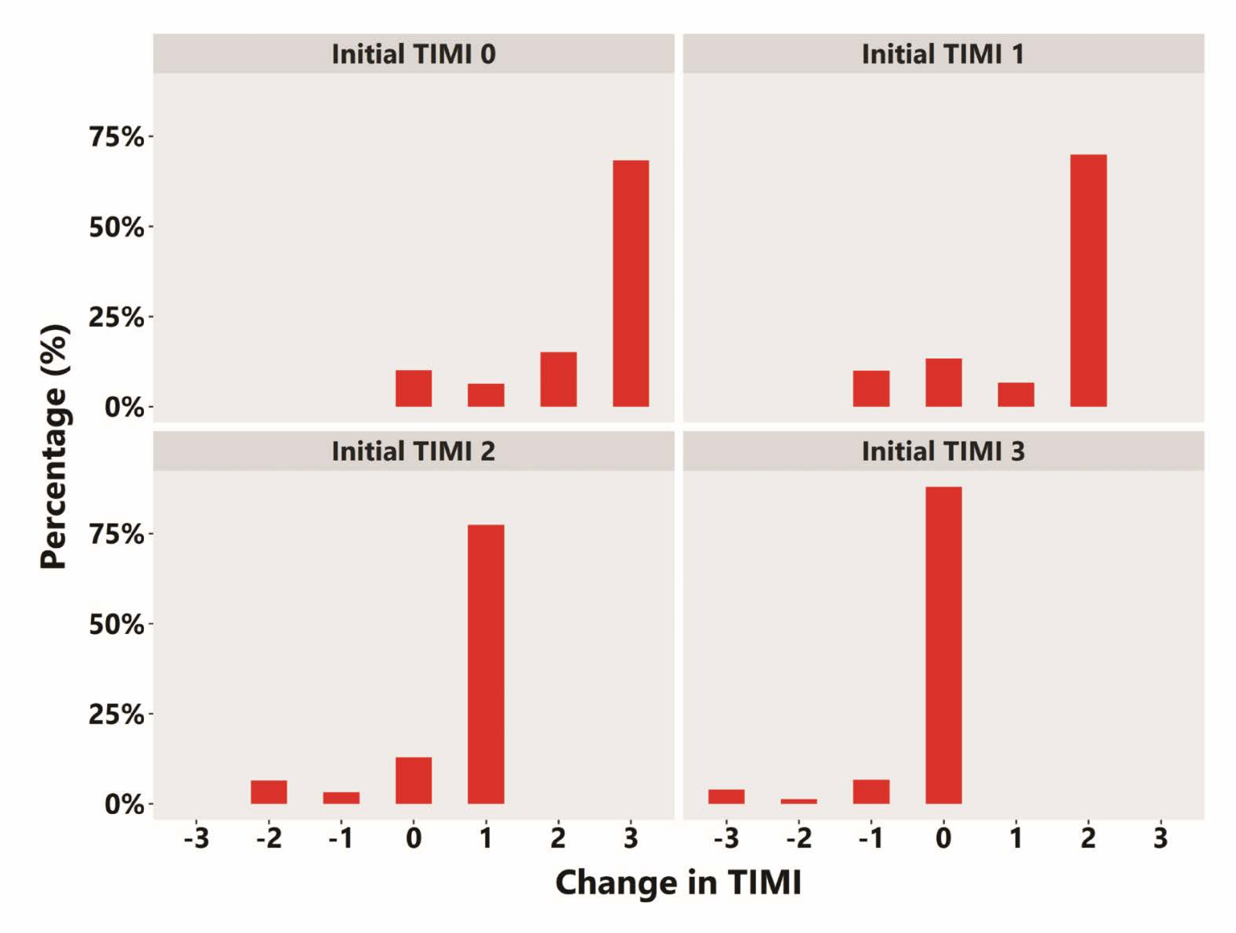

Initial TIMI 0 – n=79, Initial TIMI 1 – n=30, Initial TIMI 2 – n=31, Initial TIMI 3 – n=75

eFigure 4: A) Changes in Thrombolysis in myocardial infarction (TIMI) flow for STEMI subset of SCAD-PCI patients (n=119) and B) PCI complications in the STEMI subset of SCAD-PCI cohort (n=119)

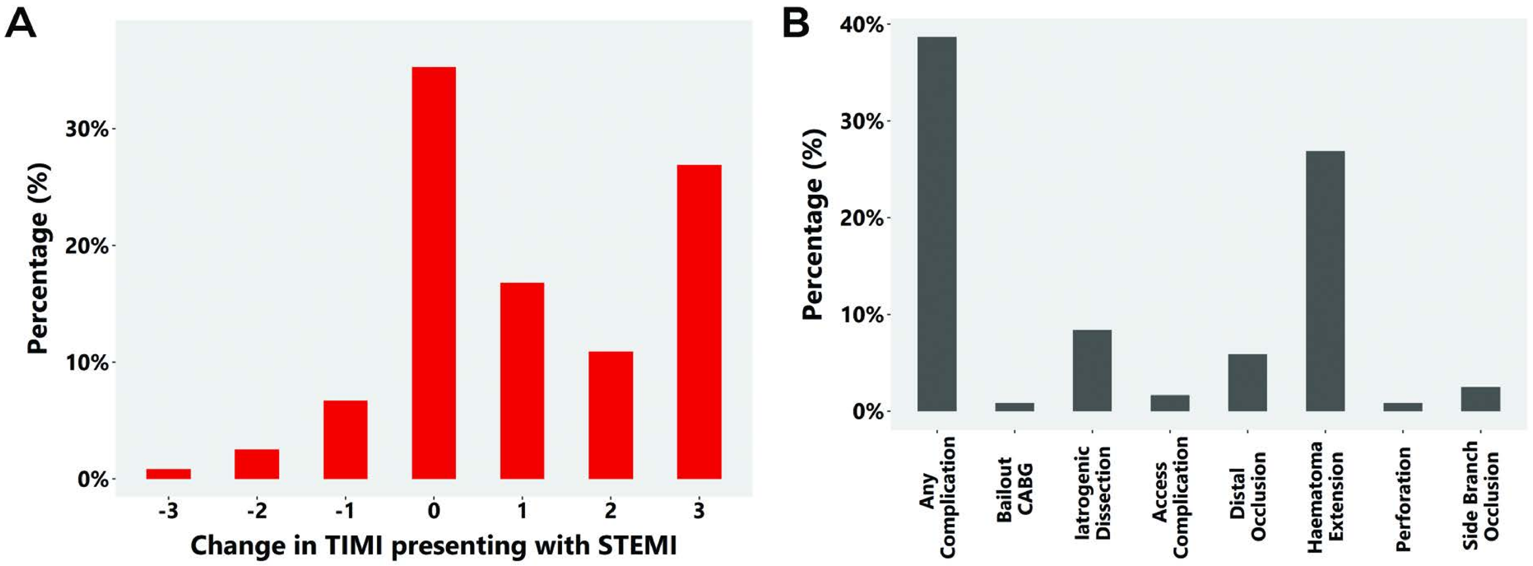

Kotecha D, *et al. Heart* 2021; 107:1398–1406. doi: 10.1136/heartjnl-2020-318914

eFigure 5: Ejection fraction by clinical management

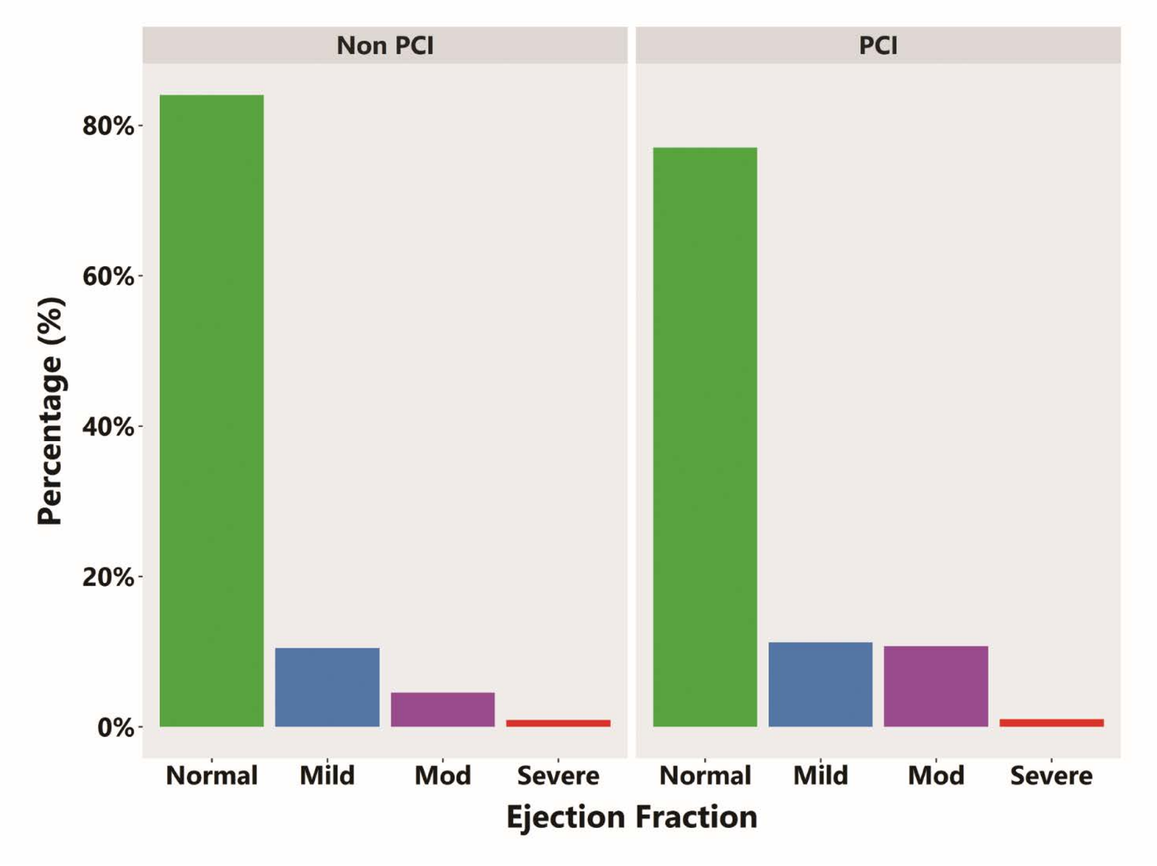

PCI: normal n=151, mild n=22, moderate n=21, severe n=2

Non-PCI: normal n=184, mild n=23, moderate n=10, severe n=2

Kotecha D, *et al. Heart* 2021; 107:1398–1406. doi: 10.1136/heartjnl-2020-318914

eFigure 6: A) Kaplan-Meier plot of major adverse cardiovascular and cerebrovascular events (n=433), and B) recurrence (n=434) by intervention in a SCAD cohort

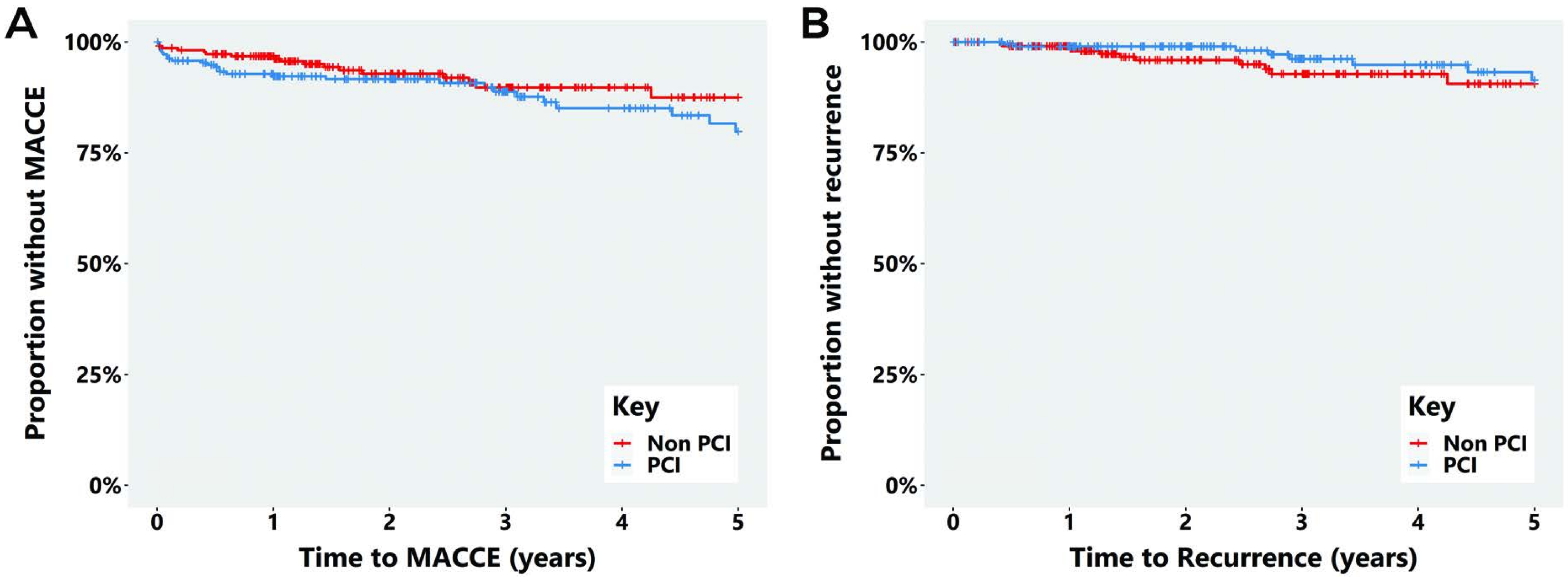

Proportional hazards model: MACCE p=0.3931, recurrence p=0.3087. Major Adverse Cardiovascular and Cerebrovascular Events (MACCE: death, stroke, myocardial infarction or revascularisation occurring at any point after discharge from the index SCAD event) and recurrence (a new angiographically confirmed SCAD occurring after discharge from the index episode and either anatomically or temporally separated from the first event). MACCE components - recurrent AMI (PCI 9.3%, 20/215, non-PCI 7.7%, 17/221); revascularisation (PCI 4.7%, non-PCI 1.4%); stroke (PCI 1.5%, non-PCI 0.7%); death (PCI 1.4%, non-PCI 0.5%))

Kotecha D, *et al. Heart* 2021; 107:1398–1406. doi: 10.1136/heartjnl-2020-318914

eTable 4: Descriptive characteristics of the SCAD cohort, by national cohort

		Spain (n=119)		Netherlands (n=39)		UK (n=278)	
**Patient Characteristics**							
Total (n %)		119	27.3%	39	8.9%	278	63.8%
Age at first SCAD event, years (median, IQR)		52	(46-58)	50	(46-54)	46	(41-52)
Ethnicity (n %)	White European	108	91.5%	>34	>87.2%	263	95.0%
	Not White European	10	8.5%	<5	<12.8%	14	5.1%
Sex (n %)	Female	110	92.4%	>34	>87.2%	261	93.9%
	Male	9	7.6%	<5	<12.8%	17	6.1%
Pregnancy status (n %)	Not pregnant (female)	>105	>95.5%	>30	>85.7%	240	88.8%
	Pregnant (female)	<5	<4.5%	<5	<14.3%	21	5.6%
Smoking (n %)	Never smoker	72	(60.5%)	29	(74.4%)	185	(66.5%)
	Ex-smoker	15	(12.6%)	5	(12.8%)	79	(28.4%)
	Current smoker	32	(26.9%)	5	(12.8%)	14	(5.0%)
Diabetes Mellitus (n %)	No	>114	(>95.8%)	>34	(>87.2%)	>273	(>98.2%)
	Yes	<5	(<4.2%)	<5	(<12.8%)	<5	(<1.8%)
Hypertension (n %)	No	90	(75.6%)	23	(59.0%)	215	(77.3%)
	Yes	29	(24.4%)	16	(41.0%)	63	(22.7%)
Dyslipidaemia (n %)	No	85	(71.4%)	33	(84.6%)	254	(91.4%)
	Yes	34	(28.6%)	6	(15.4%)	24	(8.6%)
**Clinical characteristics**							
	NSTEMI	>50	43.7%	>20	56.4%	131	47.1%

Type of myocardial infarction (n %)	STEMI	65	54.6%	14	35.9%	117	42.1%
	Cardiac arrest	<5	5.9%	<5	<12.8%	30	10.8%
Left main stem vessel affected (n %)		<5	<4.2%	<5	<13.2%	10	3.6%
Left anterior descending artery affected (n %)		63	52.9%	22	57.9%	182	65.5%
Left circumflex artery affected (n %)		38	31.9%	15	38.5%	78	28.1%
Right coronary artery affected (n %)		19	16.0%	11	28.2%	57	20.5%
AHA coronary segment involved (n %)	Proximal	23	19.3%	6	15.4%	52	18.7%
	Mid	26	21.9%	7	18.0%	97	34.9%
	Distal	27	22.7%	15	38.5%	87	31.3%
	Branch	43	36.1%	11	28.2%	42	15.1%
More than one vessel involved (n %)		9	7.6%	10	26.3%	32	11.5%
More than one segment in the vessel involved (n %)		28	23.5%	11	28.2%	104	37.4%
Tortuosity Index (median, IQR)		3	(0-5)	4	(3-6)	4	(2-6)
Yip-Saw Classification based on appearance when imaged (n %)	Type 1	24	20.2%	<5	<12.8%	27	9.7%
	Type 2	66	55.4%	30	76.9%	175	62.9%
	Type 3	12	10.1%	<5	<12.8%	26	9.4%
	Type 4	17	14.3%	<5	<12.8%	50	18.0%
Taking aspirin (n %)		110	93.2%	39	100.0%	266	97.1%
Taking DAPT (n %)		74	62.7%	32	84.2%	248	91.2%
Taking Beta-blocker (n %)		94	79.7%	26	66.7%	242	88.3%
Taking ACE inhibitors (n %)		58	49.2%	28	71.8%	218	79.3%
Taking statins (n %)		96	81.4%	25	64.1%	205	74.8%
**Intervention Details**							
Type of intervention (n %)	Conservative	59	49.6%	20	51.3%	142	51.1%

Kotecha D, *et al. Heart* 2021; 107:1398–1406. doi: 10.1136/heartjnl-2020-318914

	Stent	45	37.8%	>10	>25.6%	98	35.3%
	Balloon	>10	<8.4%	5	12.8%	27	9.7%
	Wiring	<5	<4.2%	<5	<12.8%	11	4.0%
Maximum stent diameter, mm (median, IQR)		3	(2.5-3.5)	3	(2.5-3.0)	3	(2.5-3.5)
Total number of stents (median, IQR)		2	(1-2)	2	(1-3)	2	(1-3)
Total length of stents, cm (median, IQR)		38	(25-52)	37	(26-56)	51	(32.5-68)
Proximal diameter, mm (median, IQR)		2.4	(1.95-2.70)	2.6	(2.28-3.13)	2.6	(2.20-3.10)
Length of lesion, mm (median, IQR)		32.9	(24.2-48.0)	39.7	(30.0-50.2)	38.8	(26.6-61.3)
Volume of haematoma, mm^3 (median, IQR)		42.2	(24.2-115.3)	59.6	(22.3-95.2)	61.9	(36.0-102.7)
Final TIMI grade flow (n %)	0 (No flow)	<5	<8.3%	0	0.0%	12	8.8%
	1	<5	<8.3%	<5	<26.3	7	5.2%
	2	8	13.3%	<5	<26.3	12	8.8%
	3 (Good flow)	46	76.7%	14	73.7%	105	77.2%
**Outcomes**							
Any complication (n %)		24	40.0%	6	30.0%	57	41.0%
Serious complication (n %)		10	16.7%	<5	<26.3%	15	11.0%
Time to MACCE (median, IQR)		1.10	(0.92-2.07)	1.79	(1.18-2.89)	3.01	(1.47-4.79)
MACCE (n %)		10	8.4%	5	12.8%	38	13.7%
Time to Recurrence (median, IQR)		1.11	(0.97-2.04)	1.96	(1.19-2.95)	3.09	(1.76-5.00)
Recurrence (n %)		5	4.3%	<5	<12.8%	21	7.6%

Cells with small counts of less than five have been replaced with "<5" to reduce the risk of identifying individuals from the data.

Kotecha D, *et al. Heart* 2021; 107:1398–1406. doi: 10.1136/heartjnl-2020-318914
